# Host-microbiota interaction helps to explain the bottom-up effects of climate change on a small rodent species

**DOI:** 10.1038/s41396-020-0646-y

**Published:** 2020-04-20

**Authors:** Guoliang Li, Baofa Yin, Jing Li, Jun Wang, Wanhong Wei, Daniel I. Bolnick, Xinrong Wan, Baoli Zhu, Zhibin Zhang

**Affiliations:** 1 State Key Laboratory of Integrated Pest Management, Institute of Zoology, Chinese Academy of Sciences, 100101 Beijing, China; 2 CAS Center for Excellence in Biotic Interactions, University of Chinese Academy of Sciences, 100049 Beijing, China; 3 Colleges of Bioscience and Biotechnology, Yangzhou University, Yangzhou, 225009 China; 4 Key Laboratory of Pathogenic Microbiology and Immunology, Institute of Microbiology, Chinese Academy of Sciences, 100101 Beijing, China; 5 Department of Ecology and Evolutionary Biology, University of Connecticut, 75N. Eagleville Road, Unit 3043, Storrs, CT 06269-3043 USA

**Keywords:** Population dynamics, Microbial ecology, Climate-change ecology

## Abstract

The population cycles of small rodents have puzzled biologists for centuries. There is a growing recognition of the cascading effects of climate change on the population dynamics of rodents. However, the ultimate cause for the bottom-up effects of precipitation is poorly understood, from a microbial perspective. Here, we conducted a precipitation manipulation experiment in the field, and three feeding trials with controlled diets in the laboratory. We found precipitation supplementation facilitated the recovery of a perennial rhizomatous grass (*Leymus chinensis*) species, which altered the diet composition and increase the intake of fructose and fructooligosaccharides for Brandt’s vole. Lab results showed that this nutrient shift was accompanied by the modulation of gut microbiota composition and functional pathways (especially for the degradation or biosynthesis of L-histidine). Particularly, the relative abundance of *Eubacterium hallii* was consistently increased after feeding voles with more *L. chinensis*, fructose or fructooligosaccharide. These modulations ultimately increased the production of short chain fatty acids (SCFAs) and boosted the growth of vole. This study provides evidence that the precipitation pulses cascades through the plant community to affect rodent gut microbiome. Our results highlight the importance of considering host-microbiota interaction when investigating rodent population responses to climate change.

## Introduction

Climate change is taking place at a greater pace with an increase in extreme events, including significant shifts in precipitation patterns [1]. Recent studies suggested that climate change can have a large impact on the population dynamics of many species [2–4]. Shifts in precipitation can affect plant community composition and primary productivity, especially in arid and semi-arid environments, and hence trigger cascading changes in resources availability for herbivores [5]. Resource availability, in turn, alters small mammal (e.g., rodent) fitness and population dynamics [6–8]. Although the bottom-up effect of precipitation on rodents has been well documented [9], few studies have evaluated the relationship between precipitation and rodent populations using manipulative field experiments. Likewise, field studies rarely identify the physiological mechanisms causing the bottom-up regulation of plants on rodents, or examine the role of host-microbiota interactions in this regulation.

An enormous number of microorganisms reside in the gut and play key roles in host’s energy metabolism and immune responses through the production of small molecules (e.g., short-chain fatty acids; SCFAs) [10, 11], thereby affecting the host’s growth and survival. Animal diet is one of the most influential factors contributing to the diversity and composition of gut microbiota [12–15]. For small herbivorous (e.g., rodents), especially in semi-arid environments, precipitation has a strong impact on their diet composition [7]. Shifts in precipitation patterns alter plant community composition and plant species diversity [16, 17]. For example, in Inner-Mongolian native steppe grasslands, a perennial rhizomatous grass *Leymus chinensis* (LC) has relatively low water use efficiency, so its biomass production decreases significantly when precipitation is lower [18]. However, in a wet year, LC grows rapidly and becomes the dominant species [16]. These precipitation-driven shifts in plant biomass should shift herbivore (e.g., rodent) diet. We predict that these diet shifts will have a strong influence on gut microbiota composition and microbial metabolic functioning (e.g., SFCA production) in small rodents. We also argue that changes in gut microbiota may contribute to the bottom-up effects of precipitation on rodent’s body growth and fitness. Recent advances in high-throughput sequencing of the 16S rRNA gene (amplicon sequencing) have helped us to accurately obtain the relative composition of microbial communities living in the gut [19]. Gut metagenomics and metabolomics analysis have become a well-established approach for the accurate characterization of microbial function profiling and metabolic products [20]. SCFAs are common metabolic products of carbohydrate-fermenting microbes in the distal gut, and they provide an important energy source for the host tissues and gut microbes [21]. Therefore, an integrative technology combining amplicon sequencing, metagenome sequencing and metabolomics profiling is urgently needed for gaining deeper mechanistic insights into the role of gut microbes on the bottom-up effects of precipitation.

The animal diet contains several dietary macronutrients and identifying the key nutritional components that could induce changes in gut microbiota may help to understand the process and mechanism of the bottom-up effects of shifting precipitation regimes on rodents. Previous studies indicated that an increase in dietary fructose, which influences the composition of the gut microbiota colonization by silencing the Roc protein [22], can cause dysbiosis of gut microbiota [23]. In addition, the interactions between prebiotics and gut microbiota have also received much attention in recent years [24]. Fructooligosaccharide (FOS) has been identified as a prebiotic that can exert important effects on the growth of health-promoting probiotics [25]. Given the observed differences in the content of fructose and FOS within plant species [13], we hypothesize that altered dietary composition resulting from altered precipitation may influence the dietary consumption of fructose and FOS, resulting in the distinct gut microbiome and subsequent effects on growth and health in rodents. Therefore, a controlled diet manipulation experiment (supplementation with fructose and FOS) in the laboratory is essential to verify the effects of fructose and FOS in the bottom-up processes in a rodent population.

Rainfall is considered one of the vital limiting resources in semi-arid grassland of Inner-Mongolia, where the pattern of precipitation strongly influences the net primary productivity [9]. Our modeling studies have demonstrated that the population dynamics of Brandt’s voles (*Lasiopodomys brandtii*) can be triggered by an altered pattern of precipitation and subsequent changes in vegetation (represented by normalized difference vegetation index), which is strongly associated with El Niño Southern Oscillation [9, 26]. Here, we performed a precipitation manipulation experiment using large enclosures. In the laboratory, we first conducted a feeding trial where voles were fed diets that match the different precipitation-induced diets from the field experiment. This lab trial serves to verify the effect of varied diets on voles’ growth and fitness. Previous studies reported that LC preferred by Brandt’ vole is rich in fructose and FOS [13]. Precipitation-induced facilitation on growth of LC would directly increase the dietary fructose and FOS for voles. Thus, other feeding trials were conducted to investigate the effects of dietary fructose and FOS on the gut microbiota, the SCFAs concentrations in the cecum, and the body growth of voles. We expected that (1) the bottom-up effects of precipitation on vole’s growth proceed through links between diet, gut microbiota composition, and SCFAs and (2) dietary fructose and FOS, would be key factors, helping to explain shift in microbial composition and function within voles fed with various diets under different scenarios of climate change.

## Materials and methods

### Study site

To test the effects of climate change on voles, a precipitation manipulation experiment was carried out using large enclosures in the field. The area receives an average annual precipitation of 276 mm, with approximately 68% of the annual precipitation during the rainy season (June–August) (1960–2017 climate data from China Meteorological Administration).

### Precipitation manipulation experiment

The present study used a previously established experimental system designed to study the long-term effects of human activity and climate change on the populations of Brandt’s voles (see Li et al. [27] for details). We used twelve 0.48-ha enclosures at the Research Station of Animal Ecology in Inner Mongolia. The enclosures were made of 2.4 m wide galvanized iron sheets, with 1.4 m aboveground and 1 m belowground, which prevent the entrance or escape of voles. The top of each enclosure was completely covered by raptor‐proof nylon netting (10-cm mesh size). Enclosures were randomly assigned to control, light precipitation (LP) supplementation, and medium precipitation (MP) supplementation treatments. We ran the precipitation supplementation treatments from May to September. The enclosures in the control group received only natural rainfall, and the enclosures for LP and MP treatments received a volume of well water equivalent to 10 mm and 20 mm monthly rainfall, respectively. These water treatments were designed to mimic the changing precipitation regimes expected from climate change.

Before applying the precipitation supplement, all rodents were removed from the enclosures in early April. To remove the rodents, 80 live traps were used for each enclosure. Each trap was baited with a peanut and set near the voles’ burrow entrances for 7 consecutive days. In late April, 26 new adult voles (1:1 sex ratio) were introduced into each enclosure to establish the founder population. Voles were allowed to get used to the new environment during a period of 15 days. In early May, the initial density of vole’s population each enclosure was estimated by live trapping. If Brandt’s vole abundance was lower than thirteen pairs in one enclosure, we complemented founder populations to 13 pairs with voles captured from nearby grassland. We monitored the vole population once every month from May to October using a standard capture-mark-recapture method. A total of 160 live traps were used for each live-trapping session in each enclosure. Voles were marked with a numbered metal ear tag when they were first captured. The trap location, vole’s sex, body weight, and reproductive condition were recorded before release. Based on the capture-mark-recapture assessment, young voles on their first captures (weighing around 16–18 g in early July) were chosen for the follow-up research to evaluate the effects of precipitation manipulation on vole body growth. In the last trapping session (i.e., early October), ten adult voles were randomly captured and immediately euthanized with sodium pentobarbital (1 mg/10 g body mass). The fresh feces were sampled and then frozen at −80 °C for future DNA extraction and sequencing.

Following each trapping session, in each enclosure, we randomly established five replicate plots (1 × 1 m^2^) and measured the plant community structure. The aboveground dry biomass, the relative cover, and frequency of each plant species were recorded using the methods described in Yin et al. [28]. Diet composition of the voles was determined by examining the feces with a compound microscope at ×100 magnification. To identify plant species in the feces, characteristics of the plant epidermal cells (e.g., shape and cell arrangement) were evaluated against a reference collection for all plant species in the enclosures [29]. To assess the differences in food quality of each plant species, we measured the content of fructose, FOS, crude fiber, silicon, crude protein, glucose, resistant starch, fat, and tannin following the methods described in Li et al. [13].

### Diet manipulation experiment

To assess the cascading effects of climate change on Brandt’s vole through its impact on diet composition, a grass-based diet manipulation experiment was carried out in the lab. We mainly focused on LC, *Stipa krylovii* (SK), and *Cleistogenes squarrosa* (CS) because these three species primarily dominated the plant community structure in the study area and constituted a large percentage of the vole’s food. The fresh plants were collected outside the enclosures. Plant samples were oven dried and grounded over a one-mm screen to keep all plant fragments in a uniform size. The powder of the three plants was mixed to make the food stick for voles. The specific weight ratios of LC:SK:CS for the control diet, the LP diet in the laboratory (LPL), and the MP diet in the laboratory (MPL) were 24.7%:61.9%:10.7%, 36.1%:59.1%:4.8%, and 52%:39.5%:8.5%, respectively. This composition was equivalent to the diet composition by voles in enclosures across the control, LP, and MP groups (Fig. S1). Eighteen young voles (body weight = 20 ± 2 g) were randomly assigned to the control (*n* = 6), LPL (*n* = 6), and MPL groups (*n* = 6) and fed daily with the respective diet for 1 month. To determine whether varied plant-based diets affect the metabolism of gut microbiota, we analyzed the absolute concentrations of fecal SCFA in voles fed with the control, LP, and MP diets. To assess the effects of diet composition (fructose and FOS) on vole growth, another diet manipulation experiment (i.e., dietary supplementation with fructose and FOS) was carried out in the lab. Thirty voles (mean body weight ± SD = 20 ± 1.5 g) were assigned to this experiment. The control group (*n* = 6) consumed a standard rodent chow diet, the fructose-fed groups consumed a standard rodent chow diet supplemented with 5% (F1; *n* = 6) or 10% (F2; *n* = 6) fructose, and the FOS groups consumed a standard diet supplemented with 5% (FO1; *n* = 6) or 10% (FO2; *n* = 6) FOS (Fig. S1).

All the young voles used in these experiments were captured in June from a nearby field. During the 7-day acclimatizing period in the lab environment, prior to the feeding trials, voles were supplied with standard rodent chow (Beijing Huafukang Biotechnology #2022: 3% fat, 15% crude fiber, and 14% protein) and were housed in individual cages (25.5 × 15 × 13.5 cm) under the natural photoperiod with free access to water and food. The weight of each vole and food intake were assessed once every 2 weeks after the initiation of the diet treatments. These experiments lasted for one month, and at the end of the experiment, all voles were anesthetized with sodium pentobarbital and quickly euthanized by decapitation. Fresh feces were collected and stored at −80 °C until further metagenomics and metabolomics analysis.

### Fecal microbiota transplantation (FMT)

In order to approve that the voles’ growth is mediated by the changes in microbiome, a fecal microbiota transplantation experiment was carried out. Nine adult voles (as donors) were randomly fed with control diet, LP diet, and MP diet respectively. Six to nine fresh feces pellets collected from donors were diluted in 2 mL of physiological saline, and then centrifuged at 500 *g* for 1 min, and the supernatant was used for FMT. Before transplantation, 24 young voles (as recipients, 22 ± 1 g) were treated for 4 days with 100 μL of an antibiotic cocktail (containing 100 μg/mL neomycin, 50 μg/mL streptomycin and 100 U/mL penicillin) as described previously [30]. After that, voles were allocated to three groups (*n* = 8 voles/group) to receive 100 μL of the microbiota suspension from voles fed with either control diet, LP diet or MP diet. The suspension was administered to voles by oral gavage once a week for 4 weeks. During these 4 weeks, all recipient voles were fed the same food (MP diet). Their body weights were measured once a week.

### Analysis of the sequence data

The downstream processing of the amplicon sequencing reads included: (1) merging paired-end sequences and quality control by using FLASH [31]; (2) identification and removal of the singletons and chimeras using USEARCH [32] and UCHIME [33], respectively; (3) clustering of the remaining amplicon sequences by a threshold of 97% sequence identity and comparing sequences against the GreenGenes database to generate Operational Taxonomic Units (OTUs) using USEARCH; and (4) final taxonomic assignment using RDP-classifier [34]. Sequence analysis was performed by using the Quantitative Insights Into Microbial Ecology (QIIME, version 1.9.1) software suite with custom scripts [35]. To exclude the influence of sequencing depth, the reads of each sample were normalized to 19266 reads (the minimum read number of the samples assigned to OTUs was 19266) using single_rarefaction.py script in the QIIME pipeline. Alpha diversity indices were calculated using alpha_diversity.py. Beta diversity was estimated by computing Bray-Curtis dissimilarity distances between the samples using QIIME. Linear discriminant analysis (LDA) effect size (LEfSe) (http://huttenhower.sph.harvard.edu/lefse/) was used with the default options to determine which OTUs differed across different diet compositions in the enclosure.

For shotgun sequencing data analysis, metagenomic reads were assessed using FastQC (v0.11.8) [36] and then trimmed using Trimmomatic (v0.38) [37]. In addition, Kraken [38] was employed to taxonomically profile each sample using the RefSeq [39] bacterial database from NCBI. The functional profiling of metagenomic reads was performed by HUMAnN2 [40] using DIAMOND [41] with the UniRef90 databases.

### Statistical analysis

For the field experiment, permutational multivariate analysis of variance (PERMANOVA) using Bray–Curtis distance matrices (nested adonis function in R, package “vegan”) was used to compare the differences in gut microbial community among voles between different precipitation supplementation treatments. We used a linear mixed model with enclosure specified as a random factor to test the differences in forage availability, diet composition, and gut microbial alpha indices of voles among different precipitation supplementation treatments. Random forest analysis was used to select the important features that may contribute to the differences in nutrition contents among the seven plant species [42]. For the lab experiment, Kruskal–Wallis test and pairwise Wilcoxon test were used to identify the differences in species and metabolic pathways between different diet groups [43]. Analysis of variance (ANOVA) was used to test the effect of different diets on the concentration of fecal SCFAs and body weight gains in voles. The assumption of normality was tested by using the Shapiro–Wilk test on the ANOVA residuals. The homogeneity of variance was checked using the Levene’s test and the diagnostic plots of the residuals from the fitted mode. All the analyses were carried out in R software (version 3.5.1).

## Results

### Bottom-up effects of precipitation manipulation on Brandt’s voles

**Fig. 1:**
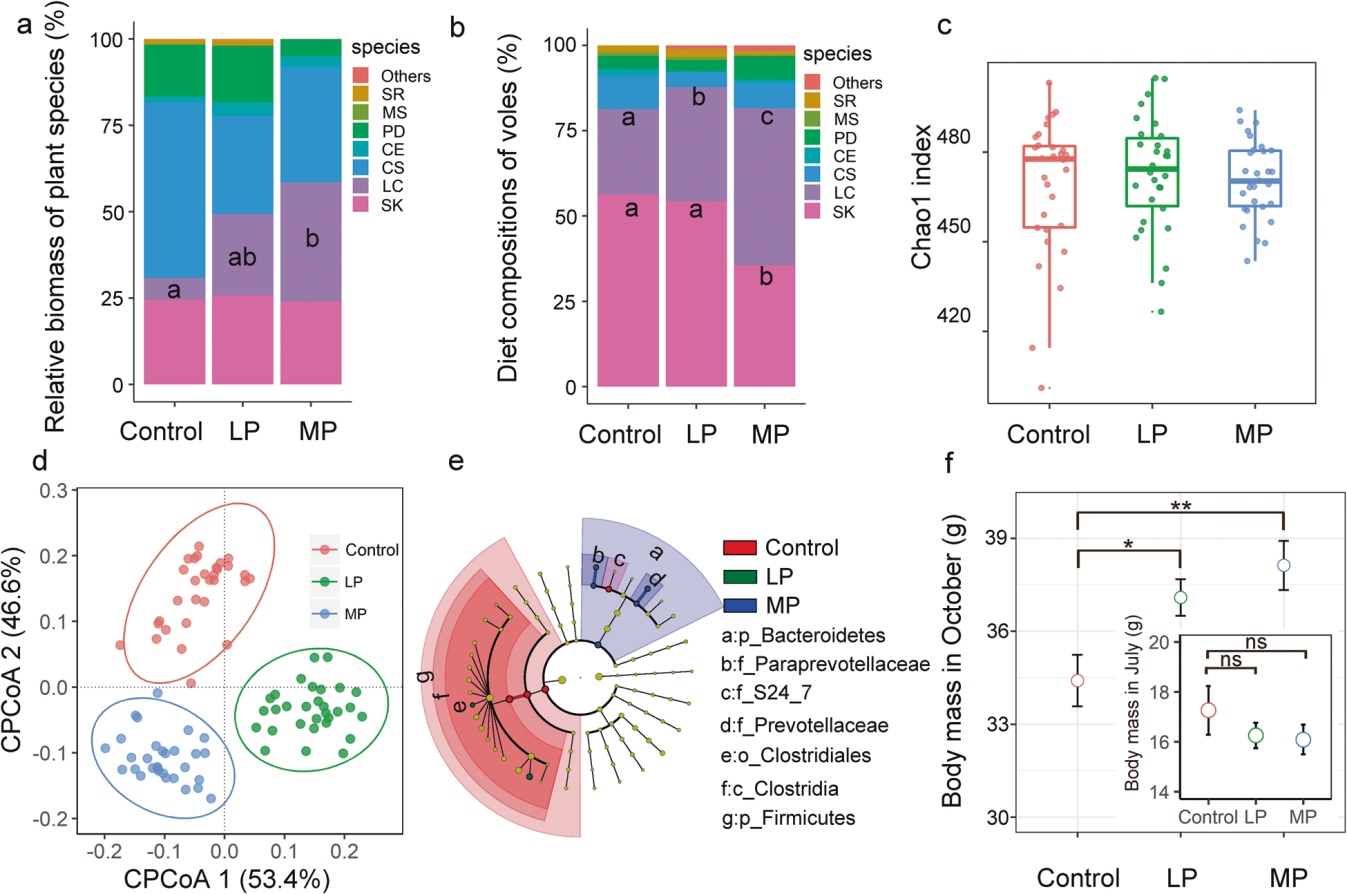
Cascading effects of precipitation manipulation on Brandt’s voles in enclosures. The roles of precipitation manipulation in forage availability and diet composition of voles (**a**, **b**). LP light precipitation supplementation group; MP medium precipitation supplementation group; SR *S. runcinata*; MS *M. sativa*; PD *P. dentosa*; CE *C. enervis*; CS *C. squarrosa*; LC *L. chinensis*; SK *S. krylovii*. Different letters indicate significant differences between the treatments (*P* < 0.05). **c** The roles of precipitation manipulation in *L. brandtii* gut microbiota community structure (Chao1 index). **d** Constrained PCoA plot of OTU-level Bray-Curtis distances between samples from control, LP, and MP. **e** Responses of *L. brandtii* gut microbiota to precipitation manipulation. Cladogram representing the bacterial biomarkers found to be significantly associated with precipitation manipulation by LEfSe (LDA > 2; *P* < 0.05). **f** Differences in *L. brandtii* body growth across different precipitation manipulation treatments from July to October (ns non-significance; *P < 0.05; **P < 0.01).

To confirm the bottom-up effects of precipitation on small mammal, we monitored the vole population and plant community in a rainfall simulation experiment. We first assessed whether the plant community differed between rainfall treatment and control. In enclosures, the plant community structure mainly comprised of species such as *C. squarrosa*, LC, SK*, Saussurea runcinata, Medicago sativa, Phlomis dentosa*, and *Carex enervis* (Fig. 1a). Moderate precipitation (MP) supplementation dramatically facilitated the overall recovery of LC (*F*_2, 9_ = 4.7, *P* = 0.04; Fig. 1a). Precipitation manipulation groups exhibited a 5.5-fold increase in the proportion of LC biomass compared with the control group (*t* = 2.8, *P* = 0.02). There was also a trend for the biomass of LC to increase in the LP supplementation group compared with the control group, although the effect was marginally non-significant (Fig. 1a). There were no significant differences in the biomass of other plant species across the precipitation treatment groups (*P* > 0.05).

We then assessed whether the vole’s diet differed between rainfall treatment and control. LC and SK were the dominant grass species in vole’s diet, accounting for more than 80% of the total food (Fig. 1b). Precipitation manipulation alters the diet composition of voles (Fig. 1b). Specifically, the proportion of LC in *L. brandtii* diets was higher in LP and MP enclosures (LP: *t* = 2.1, *P* = 0.03; MP: *t* = 5.4, *P* < 0.001; Fig. 1b), whereas the proportion of SK was lower in MP enclosures (MP, *t* = −2.3, *P* = 0.04). We found no significant differences in the abundance of other plant species in vole diets among precipitation groups (*P* > 0.05).

We further revealed extensive variation in gut microbiota of Brandt’s vole in different precipitation treatment groups by processing raw data from amplicon sequencing datasets. We obtained a total of 7875868 microbial 16S rRNA raw sequence reads (87,509 ± 13,529 reads per individual) for 90 samples from the enclosures. The sequences were then quality-filtered and clustered with 97% similarity. Consequently, we identified a total of 530 OTUs (451 ± 20 OTUs per individual) across the samples. Within these OTUs, we identified 8 phyla, 13 classes, 13 orders, 21 families, and 25 genera. The dominant phyla present were Firmicutes (87.9 ± 5 %) and Bacteroidetes (8.8 ± 2%). MP reduced the ratio of Firmicutes/Bacteroidetes from 9.7 to 6.8 compared with control group (*P* < 0.05). We found no significant effect of precipitation manipulation on OTUs-level alpha diversity metrics (observed OTU richness and Chao1; *P* > 0.05, Figs. 1c and S2). However, principal coordinates analysis (PCoA) revealed a marked distinction between samples from the control, LP, and MP groups in ordination space (PERMANOVA; *F* = 3.6, *P* = 0.0001; Fig. 1d). LEfSe analysis showed that, from phyla to genera, precipitation manipulation groups influenced *L. brandtii* gut microbiota. The taxa from the phyla *Firmicutes*, class *Clostridia*, order *Clostridiales*, and family *S24_7* were more frequently observed in *L. brandtii* gut microbiota in the control group, whereas genus *Ruminococcus* and *Marvinbryantia* were more prevalent in the *L. brandtii* gut microbiota of the LP group (Fig. 1e). The phyla *Bacteroidetes*, family *Prevotellaceae*, family *Paraprevotellaceae*, genus *Prevotella*, and genus *YRC22* were more abundant in the MP group (Fig. 1e).

To further characterize cascading effect of precipitation on vole population, here we mainly focused on the body growth of vole. In early July, the average body weight of young voles was not significantly different among the three precipitation manipulation groups (Fig. 1f). After three months of growth, body weight increased to 30–44.6 g (mean, 36.7 g). Notably, the average changes in body weight of voles in the LP and MP treatments was 7.8% and 10.8%, respectively, higher than in the control group (Fig. 1f).

### Identification of key nutrients in vole’s diet

**Fig. 2:**
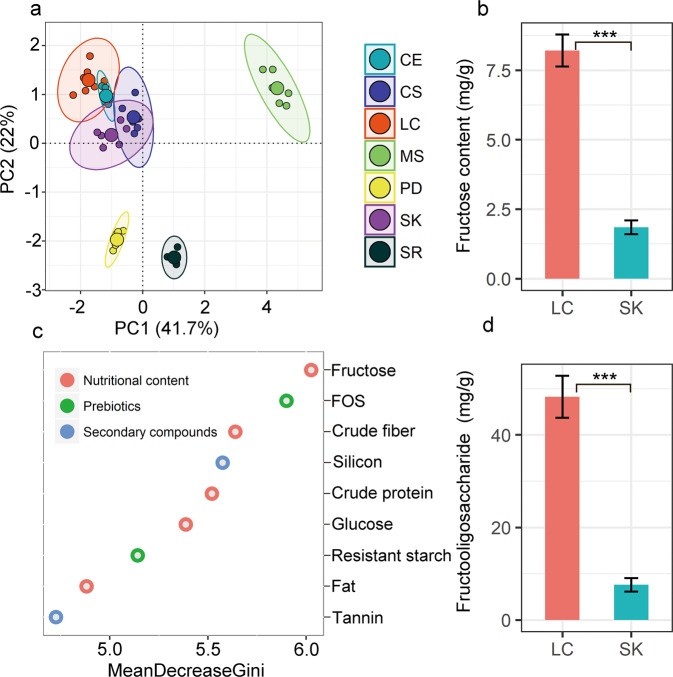
Identification of key nutrients in voleʼs diet. **a** Principal component analysis of plant nutrition distribution in seven plant species. CE: *Carex enervis*; CS *Cleistogenes squarrosa*; LC *Leymus chinensis*; MS *Medicago sativa*; PD *Phlomis dentosa*; SK *Stipa krylovii*; SR: *Saussurea runcinata*. **c** The ranking of mean decrease Gini index for the nine variables in random forests model. A higher mean decrease in Gini indicates higher variable importance. (**b**, **d**) Differences in fructose and FOS content between *L. chinensis* and *S. krylovii*.

Each plant species has its own unique nutritional composition (Fig. 2a and Table S1). PCoA analysis of seven common plant species in the vole’s diets, clearly distinguish species in ordination space based on their food quality indexes (including fructose, FOS, crude fiber, silicon, crude protein, glucose, resistant starch, fat, and tannin) included in the vole diet (*F* = 176.7, *P* < 1e−6). Samples of LC, SK, and *C. squarrosa* (belonging to family *Poaceae*) were overlapped with the samples of *C. enervis* (belonging to family *Cyperaceae*) in the ordination space (Fig. 2a). However, samples of the species belonging to family *Poaceae* and *Cyperaceae* were well separated from the samples of *S. runcinata* (family *Asteraceae*), *M. sativa* (family *Fabaceae*), and *P. dentosa* (family *Lamiaceae*). In addition, the ranking of mean decrease in Gini index for the nine nutritional indexes in the random forests model (Fig. 2c) indicated that fructose and FOS were the best variables that brought the apparent discrimination of nutrition among the seven plant species. LC and SK also had a substantial difference in the content of fructose and fructo-oliose, with fourfold higher fructose and fructo-oliose in LC compared with SK (Fig. 2b, d).

### Effect of varied plant-based diets on Brandt’s vole

**Fig. 3:**
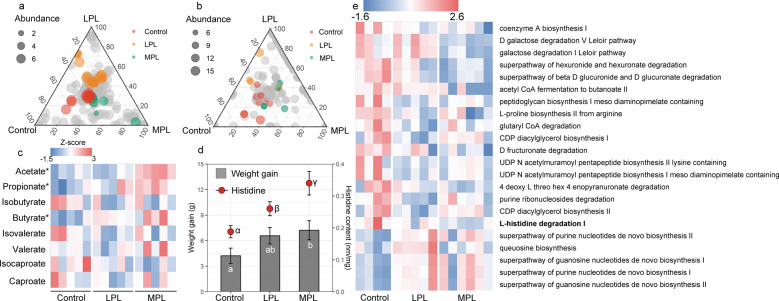
Effect of different plant-based diets on Brandt’s vole. Ternary plots showing the relative abundance of all gut microbial species (>5‱; **a**) function pathways (**b**) for control, LPL, and MPL groups. Each point corresponds to a species or one function pathway. The location of each point in the plot represents its mean relative abundance with respect to each diet group, and its size represents the mean value across all three groups. Colored points represent species or function pathways enriched in one diet group compared with the others (red in control, orange in LPL, and green in MPL samples). **c** Heat map indicating metabolite differences in short-chain fatty acids across *L. brandtii* consuming different diets. Metabolites with an asterisk represent significant differences between different diet groups. **d** Differences in weight gain and fecal histidine content across *L. brandtii* consuming different diets. **e** Marker pathways with significantly different abundances between precipitation treatment and control.

To validate the relationship between the diet change, microbiota responses and body growth, we measured the body weight and fecal amino acid concentrations and also characterized the gut microbiome using shotgun metagenomics sequencing in a diet manipulation experiment. The bacterial community of voles fed with LP diet in the lab (LPL treatment) and voles fed with MP diet in the lab (MPL treatment) was marginally different from that of voles fed with the control diet (adonis analysis: LPL, *P* = 0.1; MPL, *P* = 0.06; Fig. S3). To further quantify the number of microbial species affected by different plant diets, the distribution patterns of the dominant enriched species (at a relative abundance of >5‱) were illustrated by ternary plots among the control, LPL, and MPL groups (Fig. 3a). Of all 380 bacterial species, the number of enriched species for control, LPL, and MPL were 14, 19, and 7, respectively (Table S2), with the remaining 340 species being shared among the three diet treatments. The HUMAnN2 results suggested that the most abundant functions of gut microbiota of voles were related to adenosine ribonucleotides de novo biosynthesis, L-isoleucine and L-valine biosynthesis, and pyruvate fermentation to isobutanol (Table S2). Of all 303 microbial function pathways, we identified 26 metabolic pathways (11 pathways were enriched in control; 9 pathways were enriched in LPL; and 6 pathways were enriched in MPL; Table S3) that are differentially abundant across the control, LPL, and MPL groups (Fig. 3b). Differentially abundant metabolic functions across the three treatment diets are listed in Fig. 3e. Notably, L-histidine degradation pathways were strikingly enriched in control diet compared with LPL and MPL (*P* < 0.05).

Targeted metabolomic results showed that the fecal SCFA concentrations were significantly different in voles across the feeding treatments, and 97.7% of the fecal SCFA contained acetate, propionate, and butyrate (Fig. S4). Acetate, propionate, and butyrate concentrations in the fecal matter of voles from the MPL group were significantly higher than in the control group (acetate, *t* = 3.8, *P* = 0.002; propionate, *t* = 2.4*, P* = 0.03; butyrate, *t* = 2.6, *P* = 0.02; Fig. 3c). By contrast, isobutyrate, isovalerate, valerate, isocaproate, and caproate concentrations in the fecal matter remained unaffected (Fig. 3c). A higher weight gain was observed in voles fed with the MP diet (*P* = 0.047) for 1 month compared with the control diet (Fig. 3d). Similarly, higher fecal histidine contents were found in voles fed with LP diet or MP compared with the control diet (Fig. 3d). However, food intake across the three groups were not significantly different (*P* > 0.05).

### Effects of dietary supplementation of fructose on Brandt’s vole

**Fig. 4:**
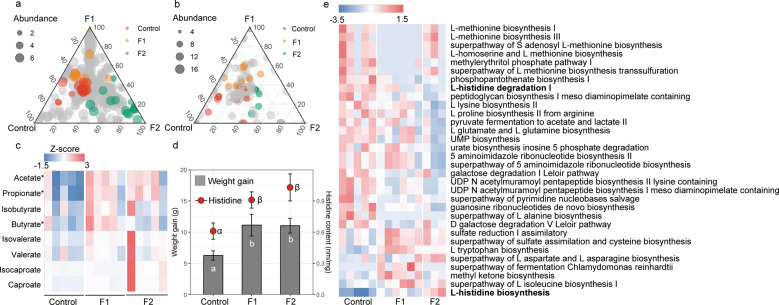
Effect of fructose supplementation on Brandt’s vole. Ternary plots showing the relative abundance of all gut microbial species (>5‱; **a**) and function pathways (**b**) for control, F1, and F2 groups. Each point corresponds to a species. The location of each point in the plot represents its mean relative abundance with respect to each diet group, and its size represents the mean value across all three groups. Colored points represent species or function pathways enriched in one diet group compared with the others (red in control, orange in F1, and green in F2 samples). **c** Heat map indicating metabolite differences in short-chain fatty acids across *L. brandtii* consuming different diets. Metabolites with an asterisk represent significant differences between different diet groups. **d** Differences in weight gain and fecal histidine content across *L. brandtii* consuming different diets. **e** Marker pathways with significantly different abundances between precipitation treatment and control.

To assess the potential roles of fructose in regulating the gut microbiome and body growth, we collected the body weight data and measured the fecal amino acid concentrations and also characterized the gut microbiome using shotgun metagenomics sequencing in a diet manipulation experiment. Different diets altered the microbial composition (Fig. S5) and functional pathways. The ternary plots showed that, among all 420 microbial species, the number of enriched species for control, F1, and F2 were 17, 12, and 15, respectively (Fig. 4a and Table S5), of the remaining 376 species shared across the three diet treatment groups. Out of the 186 function pathways, we identified 30 metabolic pathways differentially abundant across control, F1, and F2 groups. Fourteen pathways were enriched in the control, ten pathways were enriched in F1, and six pathways were enriched in F2 (Fig. 4b). Differentially abundant metabolic pathways across the three treatment diets are listed in Fig. 4e. In particular, compared with F1 and F2, the degradation pathway of histidine was markedly enriched in control diet, while the biosynthesis pathway of histidine was depleted in control diet (Fig. 4e). The concentration of acetate and propionate in the fecal samples—came from voles within the F1 group (acetate, *t* = 3.8, *P* < 0.01; propionate, *t* = 3.1, *P* < 0.01; Fig. 4c) and F2 group (acetate, *t* = 3.8, *P* < 0.01; propionate, *t* = 2.7, *P* = 0.02; Fig. 4c) were significantly higher than the control group. In addition, the concentration of butyrate in the fecal sample of the F1 group was significantly higher than the control group (*t* = 2.7, *P* = 0.02). We found no significant difference in another type of SCFA across the diet treatments (*P* > 0.05). A higher weight gain was observed for both the F1 group (*P* = 0.021) and the F2 group (*P* = 0.019) compared with the control group (Fig. 4d), although there was no significant difference in the food intake between groups (*P* > 0.05). Fecal amino acid results showed the concentration of histidine in feces was higher in F1 diet and F2 diet compared with control diet (Fig. 4d).

### Effect of dietary supplementation of FOS on Brandt’s vole

**Fig. 5:**
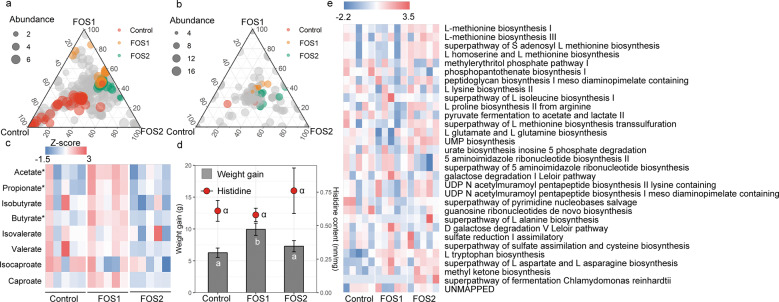
Effect of FOS supplementation on Brandt’s vole. Ternary plots showing the relative abundance of all gut microbial species (>5‱; **a**) and function pathways (**b**) for control, FOS1, and FOS2 groups. Each point corresponds to a species. The location of each point in the plot represents its mean relative abundance with respect to each diet group, and its size represents the mean value across all three groups. Colored points represent species or function pathways enriched in one diet group compared with the others (red in control, orange in FOS1, and green in FOS2 samples). **c** Heat map indicating metabolite differences in short-chain fatty acids across *L. brandtii* consuming different diets. Metabolites with an asterisk represent significant differences between different diet groups. **d** Difference in weight gain and fecal histidine content across *L. brandtii* consuming different diets. **e** Marker pathways with significantly different abundances between precipitation treatment and control.

To further assess whether the increase in dietary FOS affected the gut microbiome and body growth, we recorded the body weight and measured the amino acid concentrations and also processed raw sequencing data from fifteen shotgun sequencing datasets in a diet manipulation experiment. Different diets shaped the microbial composition (Fig. S6) and functional pathways. Out of the 367 microbial species, the number of enriched species for control, FOS1, and FOS2 were 46, 18, and 26, respectively (Table S4), with the remaining 277 species shared across diet groups. Of a total of 202 function pathways, we identified 23 metabolic pathways (three pathways were enriched in the control group; nine pathways were enriched in the FOS1 group; and 11 pathways were enriched in the FOS2 group) that were differentially abundant across the control, FOS1, and FOS2 groups (Fig. 4b). Differentially abundant metabolic functions across the three treatment diets are listed in Fig. 5e. Specifically, we did not observed differences in L-histidine degradation pathways between control and FOS supplementation groups. The concentration of acetate, propionate, and butyrate in voles’ fecal samples from the FOS1 group were significantly higher compared with the control group (acetate, *t* = 3.68, *P* < 0.01; propionate, *t* = 2.2, *P* = 0.04; and butyrate, *t* = 2.1, *P* = 0.05; Fig. 3c). However, we did not observe significant differences between FOS2 and the control group (*P* > 0.05). Interestingly, we found a negative quadratic relationship between the voles’ weight gain and the doses of FOS supplementation in the diet (Fig. 5d). This n‐shaped relationship indicated that voles with 5% FOS supplementation have more weight gain than voles with no FOS supplementation (FOS1 vs control, *P* = 0.008) and 10% FOS supplementation (FOS1 vs FOS2, *P* = 0.04; Fig. 5d). We did not find difference in fecal histidine content between control and FOS supplementation groups (Fig. 5d).

### Shared and unique microbial populations and function pathways

**Fig. 6:**
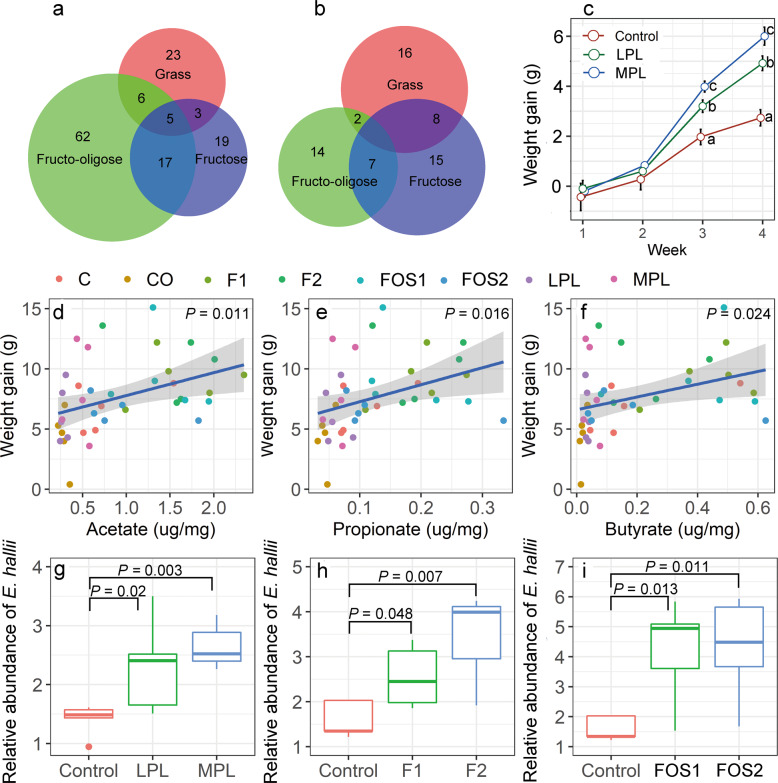
Differences in the composition and function pathways between different experiments and their relationship with body growth of voles. Venn diagrams showing the number of enriched microbial species (**a**) or enriched function pathways (**b**) shared by grass feeding, fructose addition, and FOS addition experiments. Body weight gain of voles who received feces from control, LPL and MPL donors, different letters indicate significant differences between the treatments (*P* < 0.05; **c)**. The linear relationships between short-chain fatty acid contents and *L. brandtii* weight gain (**d**–**f**). The relative abundance of *E. hallii* across different diet groups (**g**–**i**).

In the plant-based diet experiment, we reported a total of 37 enriched species. Some of the enriched species were shared with enriched species community in the fructose (21.6%) and FOS (29.7%) supplementation experiments. Notably, there were five enriched species (*Eubacterium hallii*, *Flavonifractor plautii*, *Libanicoccus massiliensis*, *Heliobacterium modesticaldum*, and *Olsenella uli*) common across the three diet experiments (Fig. 6a). There were 26 function pathways enriched in a plant-based diet experiment, of which, 30.8% and 7.7% were found with fructose and FOS supplementation, respectively (Fig. 6b).

### FMT, SCFA, and body growth

To test whether microbial changes are causal to body growth of voles in our study, a FMT experiment was carried out. Results showed that at first 2 weeks after gavage administration, voles grew slowly and no significant changes in body growth were found among control, LPL, and MPL groups (Fig. 6c). The body weight has steadily increased by the end of third week, voles who received feces from LPL and MPL donors experienced a greater weight gain than voles who received feces from control donors (Fig. 6c), although the food were all the same during the 4 weeks.

The concentrations of fecal SCFA showed a significant positive effect on the body growth of voles (acetate, *P* = 0.01; butyrate, *P* = 0.024; propionate, *P* = 0.016; Figs. 6d–f). Overall, the weight gains in voles fed with rodent chow-based diet (with/without the addition of fructose or FOS) was significantly higher than that with the plant-based diet (*t* = 2.3, *P* = 0.025). In addition, the concentrations of fecal SCFA were higher in voles fed with rodent chow-based diet than plant-based diet (acetate, *P* < 0.001; butyrate, *P* < 0.001; propionate, *P* < 0.001; Fig. S6). We found that the relative abundance of *E. hallii* was readily affected by different diet supplements. In the plant-based diet experiment, compared with the control group, the relative abundance of *E. hallii* in the LPL and MPL groups showed 64.3% and 89.3% increases, respectively (Fig. 6g). In the fructose supplementation experiment, we found that voles with dietary fructose supplementation (5 or 10%) had a significantly higher abundance of *E. hallii* than the control (Fig. 6h). Similarly, in the FOS supplementation experiment, the relative abundance of *E. hallii* in the FO1 and FO2 groups was 2.6 times higher than in the control group (Fig. 6i).

## Discussion

The importance of climate change, especially altered precipitation regimes, on the population regulation of various organisms has been widely recognized [2, 6]. It has also been well reported that gut microbiota has significant impacts on the body growth and fitness of the host animals [10, 44]. Here, we provided evidence that the bottom-up effects of changing precipitation on small rodents may be mediated by the rodents’ gut microbiota responses to dietary changes (in fructose and FOS) caused by shifting precipitation patterns.

Consistent with several previous findings [5, 7], we also reported a strong bottom-up regulation of precipitation on the vole population through changes in vegetation structure. In contrast to several previous field observational studies [16], we used a controlled precipitation manipulation experiment and found that precipitation supplementation substantially altered the plant community structure, which is consistent with previous observational studies. With increased precipitation, the diverse plant community shifted toward a LC-dominated community. Given that LC is the most preferred species by Brandt’s voles [28], we found increased consumption of LC by voles in precipitation treatment enclosures followed by a significant increase in voles’ body mass. The resulting larger body mass in the precipitation group may lead to a high survival rate in adults, which can, in turn, increase population density (Fig. S7) [45]. In addition, the previous study suggested a positive relationship between the probability of winter survival and body mass [46]. Therefore, we suggest that voles in the precipitation group may have a higher winter survival rate and may have a high rate of population growth in the following year.

Gut microbe plays an important role in body growth of juvenile animals [47]. However, for animals in a natural environment, changes in gut microbiome usually have been driven by a combination of multiple factors, including diet [13], social group, and age [48]. To understand the specific role of each factor in gut microbiota, we performed a single-factor controlled experiment in the laboratory by manipulating varying diets. Our results showed that various diets induced changes in gut microbiota composition and potential function pathways. Increased LC or fructose supplementation in the voles’ diet was associated with depleted metabolic pathways involved in L-histidine degradation I, which may explain the higher histidine concentration in feces in this study. Histidine supplementation has been reported to facilitate body growth by enhancing intestinal enzymes activities [49], increasing Zn-absorption and the thickness of the growth plate in bone [50] and forming the acetate [51]. In contrast, histidine-deficiency could decrease intestinal cell restitution through a decrease in transforming growth factor-β1 [52], which may impair gut health and body growth indirectly. This result provides some evidence that the high body mass in precipitation group was likely attributed to diet-induced changes in gut microbiota.

Different nutrient compositions in diet can modulate the gut microbiome and the function of metabolism [20]. In our study, we found nine plant species in the diet of Brandt’s voles exhibit distinct nutrient compositions (Fig. 2a and Table S1). For example, the content of fructose and FOS in LC was 4.4 and 6.3 times higher than in SK, respectively (Fig. 1b). We suggest that altered diet composition (high LC but low SK in diet) resulting from increased precipitation likely increases the total ingestion of fructose and FOS in voles, leading to increased body mass and fitness in voles. In the fructose supplementation experiment, fructose supplementation significantly increased the body growth of voles by regulating the gut microbiota composition and function pathways as well as the production of SCFAs. Fructose may stimulate the growth of selective bacterial populations, especially *Bifidobacterium*, which has been shown to improve calcium absorption with positive effects on bone turnover and body growth [21]. The metabolic process of fructose can affect the prebiotic effect of fructose. When fructose intake is low, specific transporters (GLUT5 and GLUT2) in the upper small intestine would easily absorb fructose, especially when its amount is equivalent to the amount of glucose [53]. When fructose intake is high, the amount of fructose ingested in excess of glucose will lead to a failure to completely absorb the fructose in the upper small intestine, resulting in fructose reaching the colonic lumen [53]. The unabsorbed fructose in the colon rapidly fermented to SCFAs, which exert a beneficial effect on the host [54]. These may explain the high SCFAs production and high body growth of voles in fructose supplementation groups in our study. In addition, the fructose to glucose ratio was nearly 1:1 in SK, while the fructose to glucose ratio in LC was about 2.5:1 [13]. Studies showed that fructose can be well absorbed if the consumed fructose to consumed glucose ratio was 1:1 [55]. This suggests that most fructose in LC could be unabsorbed and left to be fermented to SCFAs in the colon and increase the body mass of LC fed voles. Microbes can produce SCFAs through carbohydrate fermentation from dietary fiber, more fiber intake would increase the SCFA production. However, our previous study showed that there are no significant differences in the content of fiber among LC, SK, and *C. squarrosa* [13], so changes in SCFAs production in our study was not likely to be caused by the consumption of dietary fiber. Other study has documented that high fructose (extra 30% fructose supplementation) induced gut dysbiosis in mice and resulted in SCFAs reduction [56]. This controversial result regarding the SCFAs production may be likely caused by the different doses of fructose used in studies. Fructose has been reported to have bidirectional effects, moderate fructose supplementation could be beneficial to health, while high doses of fructose intake would be detrimental to health [57–59].

FOS are nondigestible carbohydrates and may exert a significant dose-dependent influence on the animal. For example, low-dose FOS supplementation can improve glycemic dysregulations and blood-brain-barrier integrity in mice [60], whereas high-dose FOS supplementation may lead to deteriorated glucose metabolism [61]. Consistent with these patterns, our low FOS supplementation (5%) substantially increased the body mass of voles by promoting SCFAs production, but we found no significant effects of 10% FOS supplementation. On the one hand, without interaction with gut microbiota, FOS may directly facilitate innate immune tolerance by activating host cell signaling in the intestinal epithelium [62]. On the other hand, FOS can also selectively promote the growth of some health-promoting commensal bacteria, such as *Bifidobacterium* (Fig. S8). *Bifidobacterium* would produce SCFAs from carbohydrate fermentation, which may create an acidic microenvironment to inhibit the growth of opportunistic pathogens. However, high-dose FOS supplementation may largely enhance the proliferation of *Bifidobacterium*, which also produces more lactic acid [61]. This, in turn, can hinder the growth of butyrate-producing bacteria (e.g., *Ruminococcus*) and SCFAs production [61].

SCFAs can be used as the main energy sources for both host cells and the intestinal microbiota [63] and may explain the positive relationship between SCFAs content and voles’ body growth. Indeed, we found a higher increased body mass in voles feed with rodent chow with higher acetate, propionate, and butyrate content (Fig. S6). In contrast, plant-based diet has a lower increased body mass due to lower acetate, propionate, and butyrate content. Our study also suggested a significant impact of diet manipulation (both plant-based diet and rodent chow-based diet) on the relative abundance of *E. hallii*. *E. hallii* can utilize lactate together with acetate to form butyrate [54]. In addition, *E. hallii* is also capable of metabolizing glycerol to form propionate [64]. This suggests that *E. hallii* may be recognized as a key species that may have a great impact on microbiota homeostasis. The main limitation of the study is that we did not take into account the absolute abundances during the assessment of microbial community composition, the relative abundance may not accurately reflect the actual microbiota abundance. It is urgently needed to combine the qPCR technique with high-throughput sequencing of 16S rRNA to achieve the absolute quantification of the microbiota in the future work.

## Supplementary information

supplemental material
