# Supplementary material for: Host-microbiota interaction helps to explain the bottom-up effects of climate change on a small rodent species
Source: ISME J. 2020 Apr 20;14(7):1795–808. doi: 10.1038/s41396-020-0646-y (PMC7305154; doi:10.1038/s41396-020-0646-y)
Supplement: Supplementary file 1 — supplemental material [file 41396_2020_646_MOESM1_ESM.docx]

**Supporting Information**:

**Host-microbiota interaction helps to explain the bottom-up effects of climate change on a small rodent species: A multi-omics approach**

Guoliang Li^1,5^, Baofa Yin^2^, Jing Li^3^, Jun Wang^3^, Wanhong Wei^2^, Daniel I. Bolnick^4^, Xinrong Wan^1^, Baoli Zhu^3^, Zhibin Zhang^1,5*^

^1^State Key Laboratory of Integrated Pest Management, Institute of Zoology, Chinese Academy of Sciences, Beijing 100101, China

^2^Colleges of Bioscience and Biotechnology, Yangzhou University, Yangzhou 225009, China

^3^Key Laboratory of Pathogenic Microbiology and Immunology, Institute of Microbiology, Chinese Academy of Sciences, Beijing 100101, China

^4^Department of Ecology and Evolutionary Biology 75 N. Eagleville Road, Unit 3043

University of Connecticut, Storrs, CT 06269-3043, USA

^5^CAS Center for Excellence in Biotic Interactions, University of Chinese Academy of Sciences, Beijing 100049, China

^*^To whom correspondence may be addressed: Email: [zhangzb@ioz.ac.cn](mailto:zhangzb@ioz.ac.cn)

**Running title: Climate change shapes host-microbiota interaction**

**Materials and Methods**

*Targeted metabolomic analysis*

To more precisely detect the changes in the gut metabolome of voles among different diet groups, we specially measured the concentrations of short-chain fatty acids (SCFAs; including acetate, propionate, isobutyrate, butyrate, isovalerate, valerate, isocaproate, and caproate) by using a targeted metabolomics approach as described in Zheng et al. (2013) [1]. Briefly, the fecal samples (50-100 mg each) were homogenized in acidified water containing stable isotope-labelled SCFA standards, then centrifuged at 4 °C (3000 x g, 10 min). The supernatant was withdrawn and 0.3 mL water, 0.5 mL n-propanol:pyridine mixture (3:2, v/v), and 0.1 mL propyl-chloroformate were added. After derivatization, the samples were extracted by a two-step procedure with hexane. The upper hexane layer was immediately transferred and analyzed using an Agilent 7890B gas chromatography system coupled with an Agilent 5977A Series mass spectrometric detector (MSD, Agilent Technologies Inc. CA, USA). All analyses were performed with an HP Innowax capillary column (30 m × 0.25mm × 0.25 μm, Agilent J&W Scientific, Folsom, CA, USA). One microliter of derivatives was injected in the split mode with a ratio of 10:1, and the solvent delay time was set to 2.2 min. The injection port was maintained at 260 °C, and oven temperature was programmed from 80 °C (2 min hold) at 10 °C/min to 260 °C (3 min hold). The derivatized solution was administered in splitless mode, and the acquisition was carried out in a full scan mode between 30 and 600m/z. The electron impact ionization mode was set at 70 eV.

*DNA extraction and sequencing*

DNA was extracted from the fecal samples by using a QIAamp^®^ Fast DNA Stool Mini Kit (QIAGEN^®^, www.qiagen.com). Total DNA concentration and purity were assessed with TBS-380 and NanoDrop2000, respectively. The DNA quality was examined with the 1% agarose gel electrophoresis system. The V3 hypervariable region of the 16S rRNA gene was amplified by following the methods described in Li et al. (2019) [2]. Shotgun metagenomic DNA sequencing of the fecal DNA was performed for 38 samples in the lab using an Illumina HiSeq X Ten platform with 2×150 bp paired-end (PE) reads. At least seven Gb of 150 bp PE reads per sample were generated following the manufacturer’s instructions.

*Fecal amino acids measurement*

Approximately 100 mg of feces were diluted in 20 ml of purified water and vortexed for 5 min, then incubated for 1 hour at 37°C. The supernatant was collected after centrifugation at 12000 rpm for 30min. A 1:10 dilution of the supernatant was made, and centrifuged again. Samples of 10 ul of the new supernatant was collected for derivatization reaction of amino acids. A Model U3000 DGLC UPLC analyzer (Thermo Fisher, USA) and a Waters ACCQ-TAG TMULTRA C18 chromatographic column (2.1×100 mm, 1.7 µm particle size, Waters, Ireland) were used for amino acid analysis.


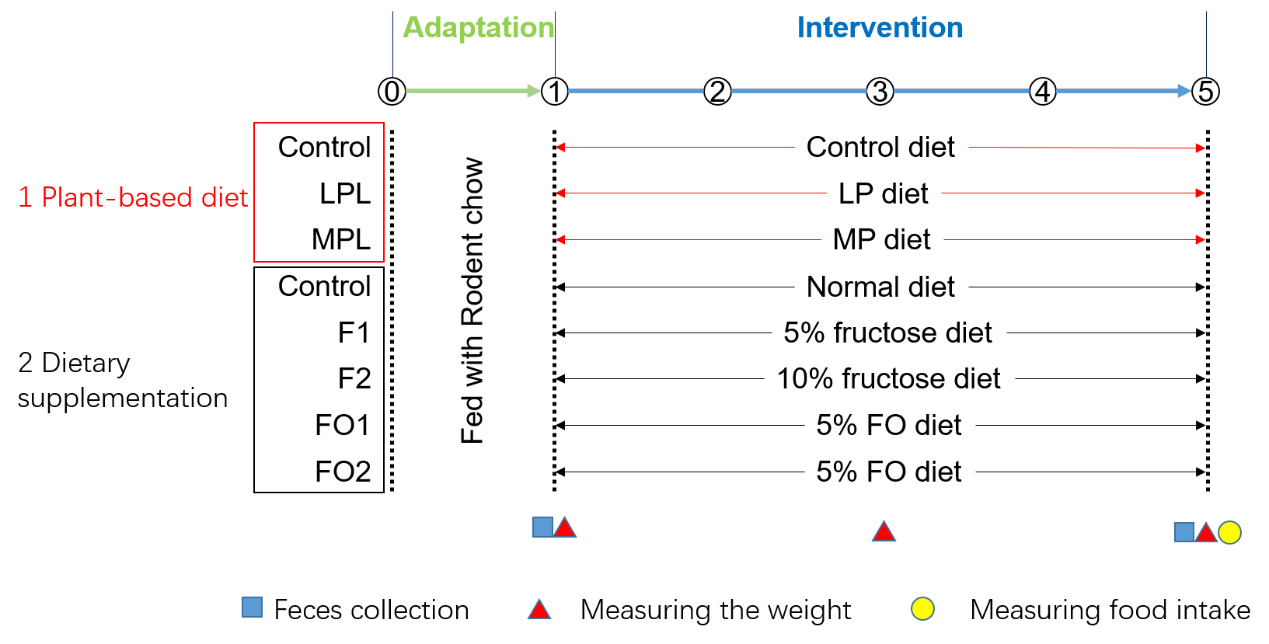


**Fig. S1** Experimental design. 48 voles were randomly assigned to eight diet groups (Control, LPL, MPL; Control, F1, F2, FO1 and FO2). During the adaptation period (first week), the voles were all fed normal diet (i.e. rodent chow). During the intervention period (from week 1 to week 5), voles were fed with control diet (the same plant diet as voles in the control enclosure), LPL diet (the same plant diet as voles in the LP enclosure), MPL diet (the same plant diet as voles in the MP enclosure), normal diet, 5% fructose supplementation diet, 10% fructose supplementation diet, 5% fructose-oligose supplementation diet, 10% fructose-oligose supplementation diet. Feces were collected before and after diet treatment on the first and fifth week. Body mass was measured at first week, third week and fifth week. The total food intake was measured at fifth week.


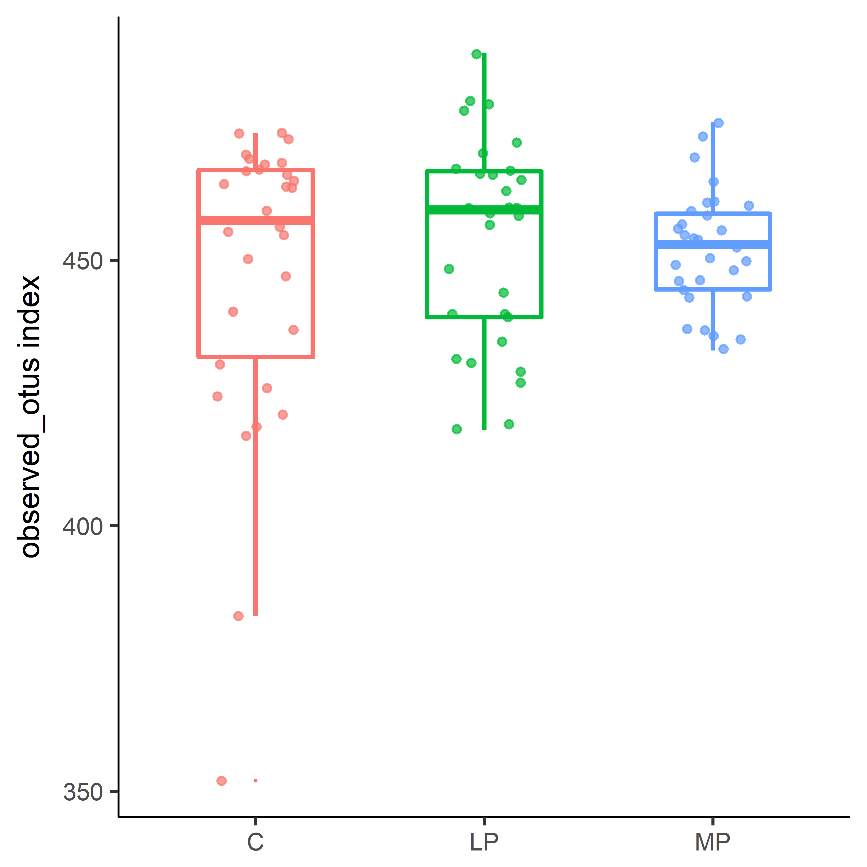


**Fig. S2** Observed richness of *L. brandtii* gut microbiota across three different precipitation group in the enclosure. C: control; LP: light precipitation; MP: medium precipitation.


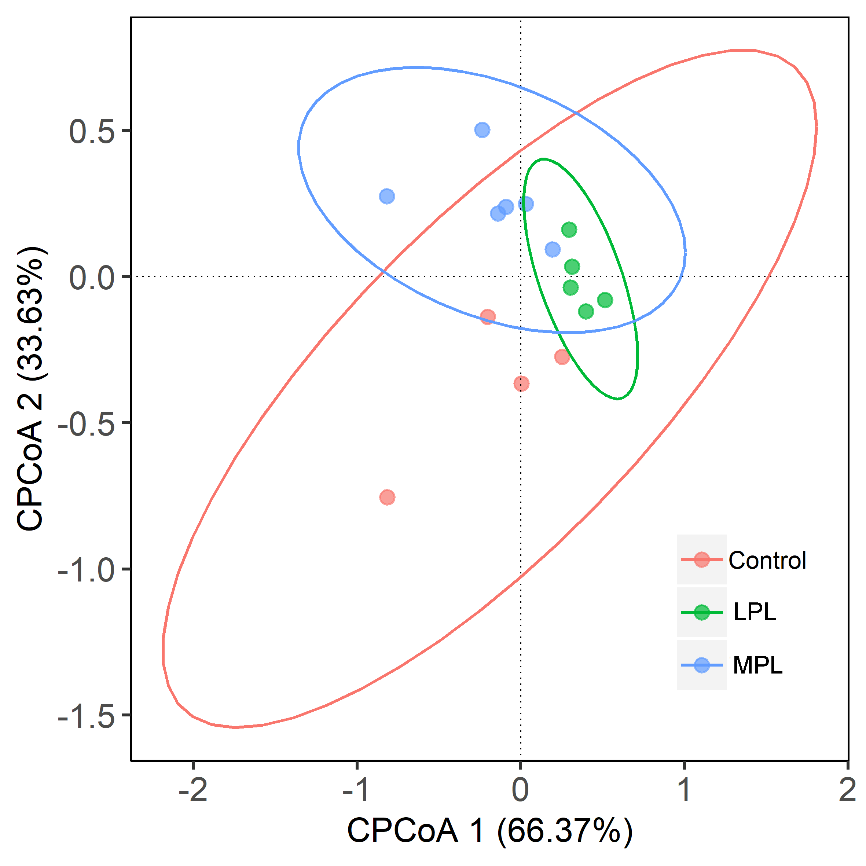


**Fig. S3**. Constrained PCoA plot of gut microbial species between samples from control, LPL and MPL group.


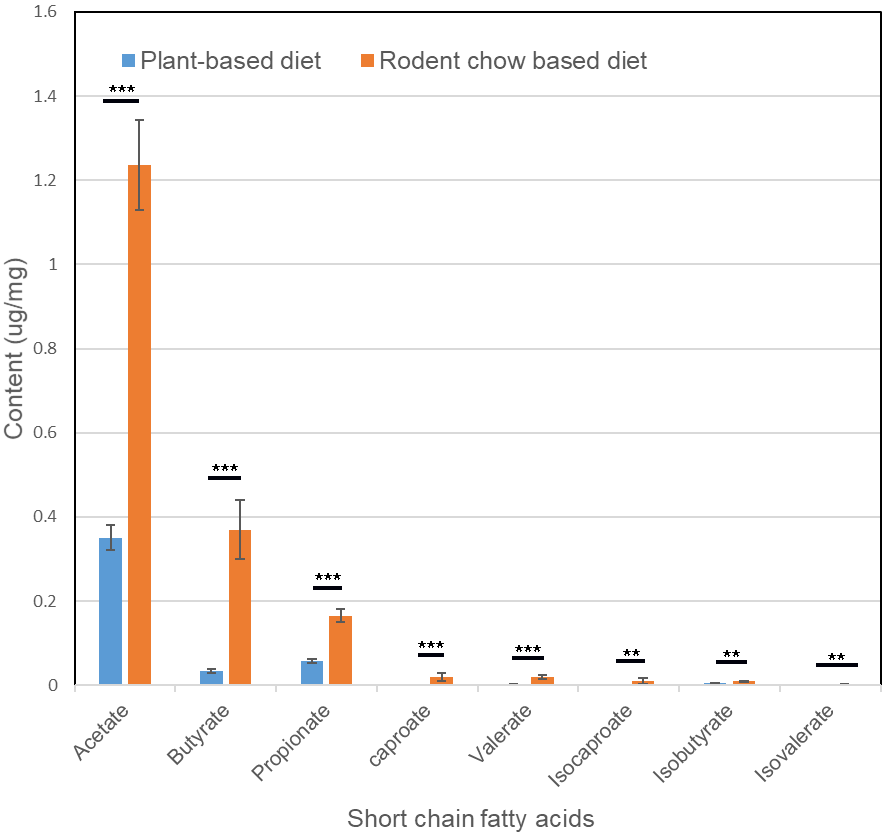


**Fig. S4** Comparison of the contents of short chain fatty acids in vole’s feces between plant-based diet experiment and rodent chow based diet experiment (**, P < 0.01; ***, P < 0.001).


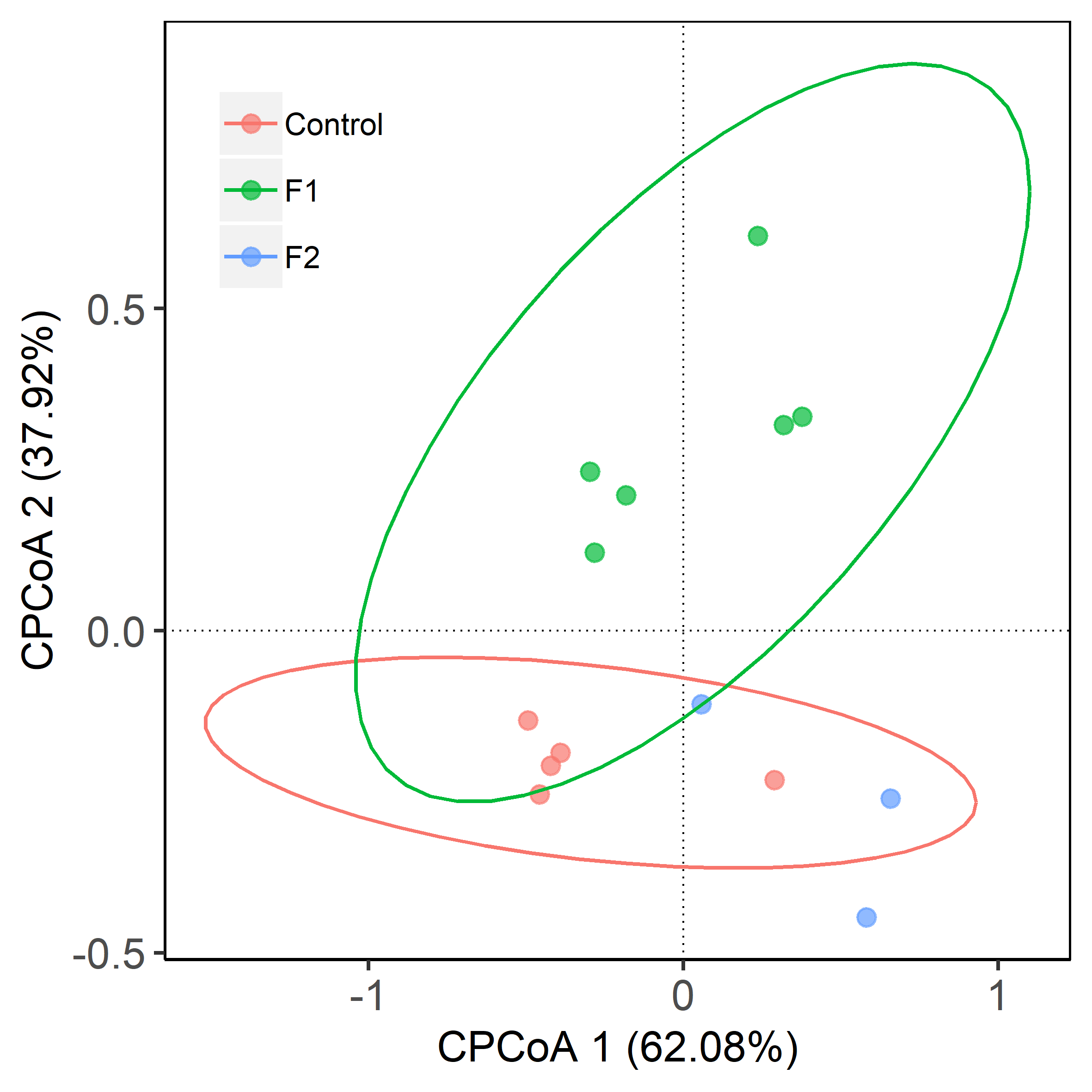


**Fig. S5**. Constrained PCoA plot of gut microbial species between samples from control, F1 and F2 group.


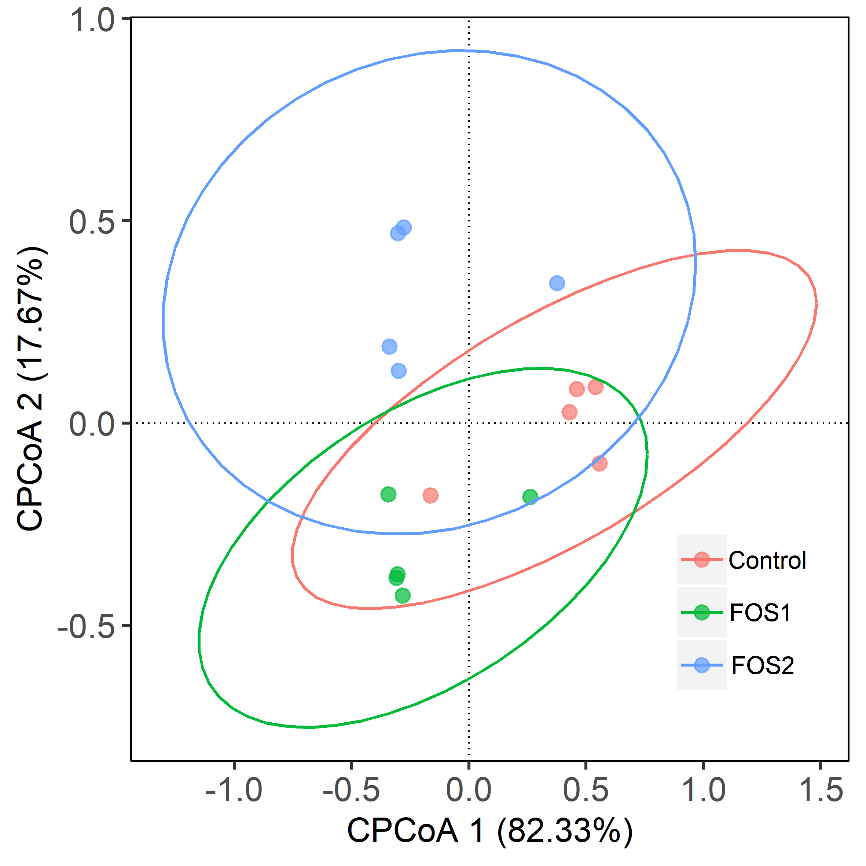


**Fig. S6**. Constrained PCoA plot of gut microbial species between samples from control, FOS1 and FOS2 group.


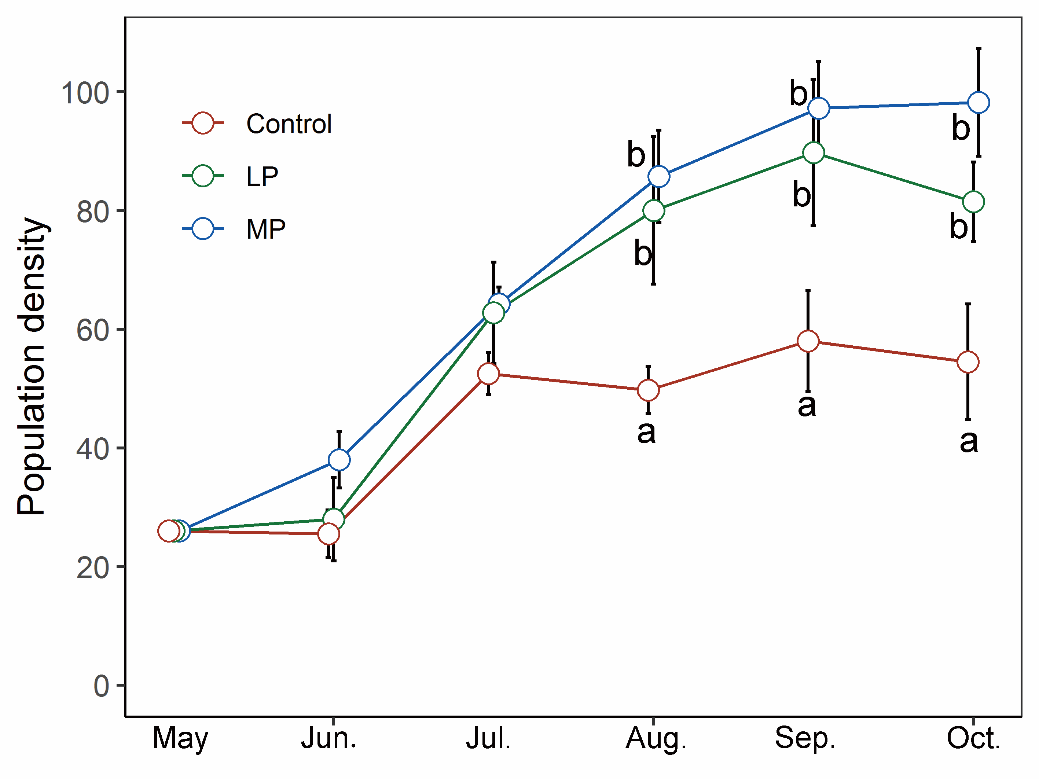


**Fig. S7** Population density (mean±se) in field enclosures treated with different precipitation supplementation. LP: light precipitation supplementation; MP: moderate precipitation supplementation. Different letters indicate significant differences between the treatments (P < 0.05).


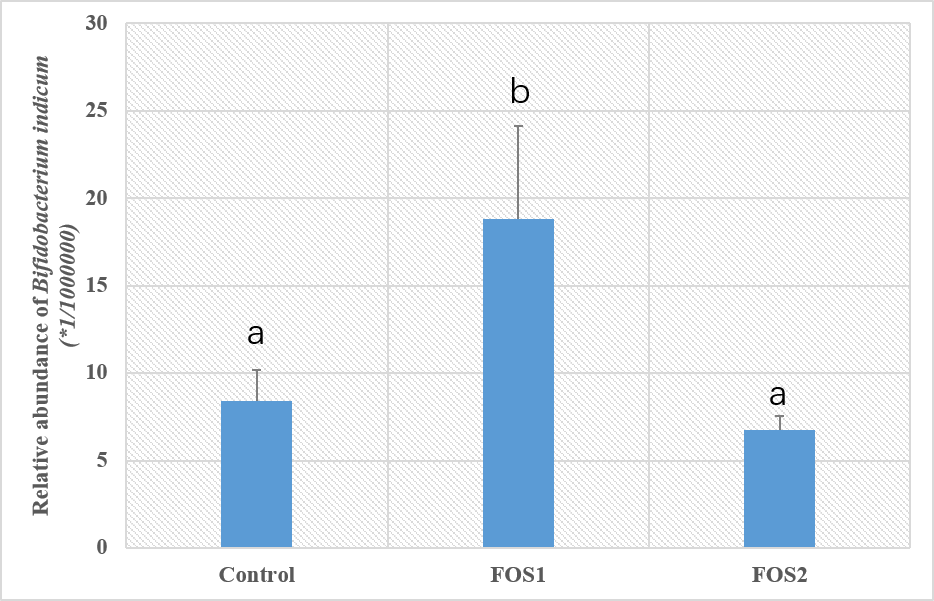


**Fig. S8** Difference in relative abundance of *Bifidobacterium indicum* between different diets. Different letters indicated significant difference. Control: fed voles with rodent chow; FOS1: rodent chow supplemented with 5% FOS; FOS2: rodent chow supplemented with 10% FOS.

| Pairs | F.Model | R^2^ | p.value |
| --- | --- | --- | --- |
| SK vs LC | 86.55175 | 0.887239 | 0.002 |
| SK vs CS | 187.8093 | 0.949446 | 0.006 |
| SK vs PD | 130.3952 | 0.928772 | 0.004 |
| SK vs SR | 222.6407 | 0.957015 | 0.004 |
| SK vs MS | 124.8548 | 0.925846 | 0.003 |
| SK vs CE | 23.71117 | 0.703362 | 0.002 |
| LC vs CS | 50.65689 | 0.821593 | 0.001 |
| LC vs PD | 72.5491 | 0.868341 | 0.002 |
| LC vs SR | 230.9617 | 0.954538 | 0.004 |
| LC vs MS | 101.0816 | 0.901857 | 0.001 |
| LC vs CE | 211.0659 | 0.950465 | 0.002 |
| CS vs PD | 300.3302 | 0.967776 | 0.004 |
| CS vs SR | 333.5324 | 0.970891 | 0.005 |
| CS vs MS | 164.526 | 0.942702 | 0.004 |
| CS vs CE | 765.4493 | 0.987104 | 0.004 |
| PD vs SR | 303.57 | 0.968109 | 0.006 |
| PD vs MS | 81.80172 | 0.89107 | 0.005 |
| PD vs CE | 812.5563 | 0.987843 | 0.004 |
| SR vs MS | 166.5413 | 0.943356 | 0.003 |
| SR vs CE | 1143.735 | 0.991333 | 0.001 |
| MS vs CE | 277.3248 | 0.965196 | 0.003 |

**Table S1**. Results of pairwise adonis analysis for comparison of nutritional composition among 7 plant species. SR: *S. runcinata*; MS: *M. sativa*; PD: *P. dentosa*; CE: *C. enervis*; CS: *C. squarrosa*; LC: *L. chinensis*; SK: *S. krylovii*.

| Enriched species | Groups | Experiment |
| --- | --- | --- |
| s_Actinosynnema mirum | MPL | Plant-based diet |
| s_Candidatus Filomicrobium marinum | MPL | Plant-based diet |
| s_Eubacterium hallii | MPL | Plant-based diet |
| s_Flavonifractor plautii | MPL | Plant-based diet |
| s_Libanicoccus massiliensis | MPL | Plant-based diet |
| s_Shewanella frigidimarina | MPL | Plant-based diet |
| s_Citrobacter werkmanii | LPL | Plant-based diet |
| s_Corynebacterium simulans | LPL | Plant-based diet |
| s_Granulicella mallensis | LPL | Plant-based diet |
| s_Granulicella tundricola | LPL | Plant-based diet |
| s_Mycobacterium avium | LPL | Plant-based diet |
| s_Mycobacterium colombiense | LPL | Plant-based diet |
| s_Pseudomonas alcaligenes | LPL | Plant-based diet |
| s_Pseudomonas azotoformans | LPL | Plant-based diet |
| s_Pseudomonas cedrina | LPL | Plant-based diet |
| s_Pseudomonas corrugata | LPL | Plant-based diet |
| s_Pseudomonas mediterranea | LPL | Plant-based diet |
| s_Pseudomonas orientalis | LPL | Plant-based diet |
| s_Pseudomonas protegens | LPL | Plant-based diet |
| s_Pseudomonas rhodesiae | LPL | Plant-based diet |
| s_Streptomyces puniciscabiei | LPL | Plant-based diet |
| s_Treponema caldarium | LPL | Plant-based diet |
| s_Treponema pedis | LPL | Plant-based diet |
| s_Treponema putidum | LPL | Plant-based diet |
| s_Christensenella massiliensis | Control | Plant-based diet |
| s_Clostridium beijerinckii | Control | Plant-based diet |
| s_Clostridium botulinum | Control | Plant-based diet |
| s_Desulfotomaculum ruminis | Control | Plant-based diet |
| s_Faecalibaculum rodentium | Control | Plant-based diet |
| s_Heliobacterium modesticaldum | Control | Plant-based diet |
| s_Olsenella uli | Control | Plant-based diet |
| s_Peptoclostridium acidaminophilum | Control | Plant-based diet |
| s_Slackia heliotrinireducens | Control | Plant-based diet |
| s_Sphaerochaeta coccoides | Control | Plant-based diet |
| s_Sphaerochaeta globosa | Control | Plant-based diet |
| s_Sphaerochaeta pleomorpha | Control | Plant-based diet |
| s_Thermoclostridium stercorarium | Control | Plant-based diet |

**Table S2** Enriched species in Brandt’s vole gut microbiota among control, LPL and MPL groups. LPL and MPL represents feeding voles in the laboratory with diet of light precipitation group and diet of medium precipitation group respectively in the enclosure.

**Table S3** Top ten of most abundant function annotated by HUMAnN2 against UniRef90 database for experiments in the lab. Relative abundances were determined normalizing by the total number of reads.

| Pathway | Relative abundance | Experiment |
| --- | --- | --- |
| Adenosine ribonucleotides de novo biosynthesis | 0.000815898 | Feeding grass |
| L-isoleucine biosynthesis I (from threonine) | 0.000701107 | Feeding grass |
| Pyruvate fermentation to isobutanol (engineered) | 0.000701107 | Feeding grass |
| L-valine biosynthesis | 0.000701107 | Feeding grass |
| tRNA charging | 0.000571874 | Feeding grass |
| Queuosine biosynthesis | 0.00046189 | Feeding grass |
| 5-aminoimidazole ribonucleotide biosynthesis II | 0.000450869 | Feeding grass |
| Superpathway of 5-aminoimidazole ribonucleotide biosynthesis | 0.000450869 | Feeding grass |
| Superpathway of branched amino acid biosynthesis | 0.000412685 | Feeding grass |
| Glycolysis IV (plant cytosol) | 0.000399082 | Feeding grass |
|  |  |  |
| Adenosine ribonucleotides de novo biosynthesis | 0.000795355 | Feeding FO |
| L-isoleucine biosynthesis I (from threonine) | 0.000714641 | Feeding FO |
| Pyruvate fermentation to isobutanol (engineered) | 0.000714641 | Feeding FO |
| L-valine biosynthesis | 0.000714641 | Feeding FO |
| tRNA charging | 0.000562239 | Feeding FO |
| Queuosine biosynthesis | 0.000489837 | Feeding FO |
| 5-aminoimidazole ribonucleotide biosynthesis II | 0.00046043 | Feeding FO |
| Superpathway of 5-aminoimidazole ribonucleotide biosynthesis | 0.00046043 | Feeding FO |
| Glycolysis IV (plant cytosol) | 0.000401002 | Feeding FO |
| Superpathway of branched amino acid biosynthesis | 0.000338694 | Feeding FO |
|  |  |  |
| Adenosine ribonucleotides de novo biosynthesis | 0.000797773 | Feeding F |
| L-isoleucine biosynthesis I (from threonine) | 0.000721136 | Feeding F |
| Pyruvate fermentation to isobutanol (engineered) | 0.000721136 | Feeding F |
| L-valine biosynthesis | 0.000721136 | Feeding F |
| tRNA charging | 0.000571648 | Feeding F |
| Queuosine biosynthesis | 0.00047033 | Feeding F |
| 5-aminoimidazole ribonucleotide biosynthesis II | 0.000462622 | Feeding F |
| Superpathway of 5-aminoimidazole ribonucleotide biosynthesis | 0.000462622 | Feeding F |
| Glycolysis IV (plant cytosol) | 0.000387388 | Feeding F |
| Superpathway of branched amino acid biosynthesis | 0.000356384 | Feeding F |

**Table S4** Enriched species in Brandt’s vole gut microbiota among control, F1 and F2 groups. F1 and F2 represents dietary supplementation of 5% and 10% fructose respectively in the laboratory.

| Enriched species | Groups | Experiment |
| --- | --- | --- |
| s__Actinoalloteichus hoggarensis | F2 | Dietary supplementation of fructose |
| s__Borrelia parkeri | F2 | Dietary supplementation of fructose |
| s__Candidatus Borrelia tachyglossi | F2 | Dietary supplementation of fructose |
| s__Draconibacterium orientale | F2 | Dietary supplementation of fructose |
| s__Enterobacter bugandensis | F2 | Dietary supplementation of fructose |
| s__Escherichia fergusonii | F2 | Dietary supplementation of fructose |
| s__Eubacterium hallii | F2 | Dietary supplementation of fructose |
| s__Hymenobacter swuensis | F2 | Dietary supplementation of fructose |
| s__Paludisphaera borealis | F2 | Dietary supplementation of fructose |
| s__Prevotella intermedia | F2 | Dietary supplementation of fructose |
| s__Singulisphaera acidiphila | F2 | Dietary supplementation of fructose |
| s__Tessaracoccus aquimaris | F2 | Dietary supplementation of fructose |
| s__Treponema caldarium | F2 | Dietary supplementation of fructose |
| s__Treponema pedis | F2 | Dietary supplementation of fructose |
| s__Treponema putidum | F2 | Dietary supplementation of fructose |
| s__Bacillus cytotoxicus | F1 | Dietary supplementation of fructose |
| s__Bacillus krulwichiae | F1 | Dietary supplementation of fructose |
| s__Bacillus mycoides | F1 | Dietary supplementation of fructose |
| s__Bacillus pseudofirmus | F1 | Dietary supplementation of fructose |
| s__Bacillus pseudomycoides | F1 | Dietary supplementation of fructose |
| s__Bacillus thuringiensis | F1 | Dietary supplementation of fructose |
| s__Jeotgalibaca dankookensis | F1 | Dietary supplementation of fructose |
| s__Kocuria turfanensis | F1 | Dietary supplementation of fructose |
| s__Paenibacillus ihbetae | F1 | Dietary supplementation of fructose |
| s__Paenibacillus riograndensis | F1 | Dietary supplementation of fructose |
| s__Paenibacillus stellifer | F1 | Dietary supplementation of fructose |
| s__Prevotella denticola | F1 | Dietary supplementation of fructose |
| s__Acidaminococcus fermentans | Control | Dietary supplementation of fructose |
| s__Adlercreutzia equolifaciens | Control | Dietary supplementation of fructose |
| s__Akkermansia muciniphila | Control | Dietary supplementation of fructose |
| s__Alistipes finegoldii | Control | Dietary supplementation of fructose |
| s__Alistipes shahii | Control | Dietary supplementation of fructose |
| s__Bacillus cereus | Control | Dietary supplementation of fructose |
| s__Faecalibacterium prausnitzii | Control | Dietary supplementation of fructose |
| s__Flavonifractor plautii | Control | Dietary supplementation of fructose |
| s__Heliobacterium modesticaldum | Control | Dietary supplementation of fructose |
| s__Libanicoccus massiliensis | Control | Dietary supplementation of fructose |
| s__Limnochorda pilosa | Control | Dietary supplementation of fructose |
| s__Megasphaera elsdenii | Control | Dietary supplementation of fructose |
| s__Olsenella uli | Control | Dietary supplementation of fructose |
| s__Thermaerobacter marianensis | Control | Dietary supplementation of fructose |
| s__Treponema azotonutricium | Control | Dietary supplementation of fructose |
| s__Treponema denticola | Control | Dietary supplementation of fructose |
| s__Treponema primitia | Control | Dietary supplementation of fructose |

**Table S5** Enriched species in Brandt’s vole gut microbiota among control, FOS1 and FOS2 groups. FO1 and FO2 represents dietary supplementation of 5% and 10% fructose-oligose respectively in the laboratory.

| Enriched species | Groups | Experiment |
| --- | --- | --- |
| s__Citrobacter braakii | FO2 | Dietary supplementation of FOS |
| s__Citrobacter freundii | FO2 | Dietary supplementation of FOS |
| s__Enterobacter cancerogenus | FO2 | Dietary supplementation of FOS |
| s__Enterobacter kobei | FO1 | Dietary supplementation of FOS |
| s__Enterobacter ludwigii | FO2 | Dietary supplementation of FOS |
| s__Enterobacter roggenkampii | FO2 | Dietary supplementation of FOS |
| s__Escherichia albertii | FO2 | Dietary supplementation of FOS |
| s__Escherichia coli | FO2 | Dietary supplementation of FOS |
| s__Eubacterium hallii | FO2 | Dietary supplementation of FOS |
| s__Klebsiella oxytoca | FO2 | Dietary supplementation of FOS |
| s__Klebsiella pneumoniae | FO2 | Dietary supplementation of FOS |
| s__Kosakonia cowanii | FO2 | Dietary supplementation of FOS |
| s__Pseudomonas alkylphenolica | FO2 | Dietary supplementation of FOS |
| s__Pseudomonas amygdali | FO2 | Dietary supplementation of FOS |
| s__Pseudomonas antarctica | FO2 | Dietary supplementation of FOS |
| s__Pseudomonas azotoformans | FO2 | Dietary supplementation of FOS |
| s__Pseudomonas cerasi | FO2 | Dietary supplementation of FOS |
| s__Pseudomonas corrugata | FO2 | Dietary supplementation of FOS |
| s__Pseudomonas frederiksbergensis | FO2 | Dietary supplementation of FOS |
| s__Pseudomonas libanensis | FO2 | Dietary supplementation of FOS |
| s__Pseudomonas orientalis | FO2 | Dietary supplementation of FOS |
| s__Pseudomonas reinekei | FO2 | Dietary supplementation of FOS |
| s__Pseudomonas savastanoi | FO2 | Dietary supplementation of FOS |
| s__Pseudomonas synxantha | FO2 | Dietary supplementation of FOS |
| s__Pseudomonas trivialis | FO2 | Dietary supplementation of FOS |
| s__Saccharospirillum mangrovi | FO2 | Dietary supplementation of FOS |
| s__Sphingobium chlorophenolicum | FO2 | Dietary supplementation of FOS |
| s__Bacteroides caecimuris | FO1 | Dietary supplementation of FOS |
| s__Bacteroides cellulosilyticus | FO1 | Dietary supplementation of FOS |
| s__Borrelia parkeri | FO1 | Dietary supplementation of FOS |
| s__Borreliella burgdorferi | FO1 | Dietary supplementation of FOS |
| s__Candidatus Blochmannia floridanus | FO1 | Dietary supplementation of FOS |
| s__Candidatus Borrelia tachyglossi | FO1 | Dietary supplementation of FOS |
| s__Candidatus Purcelliella pentastirinorum | FO1 | Dietary supplementation of FOS |
| s__Paludisphaera borealis | FO1 | Dietary supplementation of FOS |
| s__Pseudomonas cedrina | FO1 | Dietary supplementation of FOS |
| s__Pseudomonas entomophila | FO1 | Dietary supplementation of FOS |
| s__Pseudomonas monteilii | FO1 | Dietary supplementation of FOS |
| s__Pseudomonas plecoglossicida | FO1 | Dietary supplementation of FOS |
| s__Pseudomonas poae | FO1 | Dietary supplementation of FOS |
| s__Pseudomonas pseudoalcaligenes | FO1 | Dietary supplementation of FOS |
| s__Selenomonas ruminantium | FO1 | Dietary supplementation of FOS |
| s__Selenomonas sputigena | FO1 | Dietary supplementation of FOS |
| s__Singulisphaera acidiphila | FO1 | Dietary supplementation of FOS |
| s__Adlercreutzia equolifaciens | Control | Dietary supplementation of FOS |
| s__Akkermansia muciniphila | Control | Dietary supplementation of FOS |
| s__Alistipes finegoldii | Control | Dietary supplementation of FOS |
| s__Alistipes shahii | Control | Dietary supplementation of FOS |
| s__Aminomonas paucivorans | Control | Dietary supplementation of FOS |
| s__Collinsella aerofaciens | Control | Dietary supplementation of FOS |
| s__Corallococcus coralloides | Control | Dietary supplementation of FOS |
| s__Cupriavidus taiwanensis | Control | Dietary supplementation of FOS |
| s__Dehalobacterium formicoaceticum | Control | Dietary supplementation of FOS |
| s__Desulfocapsa sulfexigens | Control | Dietary supplementation of FOS |
| s__Desulfovibrio africanus | Control | Dietary supplementation of FOS |
| s__Ehrlichia canis | Control | Dietary supplementation of FOS |
| s__Ehrlichia chaffeensis | Control | Dietary supplementation of FOS |
| s__Faecalibacterium prausnitzii | Control | Dietary supplementation of FOS |
| s__Faecalibaculum rodentium | Control | Dietary supplementation of FOS |
| s__Flavonifractor plautii | Control | Dietary supplementation of FOS |
| s__Gordonibacter massiliensis | Control | Dietary supplementation of FOS |
| s__Gordonibacter pamelaeae | Control | Dietary supplementation of FOS |
| s__Gordonibacter urolithinfaciens | Control | Dietary supplementation of FOS |
| s__Heliobacterium modesticaldum | Control | Dietary supplementation of FOS |
| s__Hungateiclostridium saccincola | Control | Dietary supplementation of FOS |
| s__Hymenobacter sedentarius | Control | Dietary supplementation of FOS |
| s__Libanicoccus massiliensis | Control | Dietary supplementation of FOS |
| s__Limnochorda pilosa | Control | Dietary supplementation of FOS |
| s__Megasphaera elsdenii | Control | Dietary supplementation of FOS |
| s__Methylocystis bryophila | Control | Dietary supplementation of FOS |
| s__Neorhizobium galegae | Control | Dietary supplementation of FOS |
| s__Niveispirillum cyanobacteriorum | Control | Dietary supplementation of FOS |
| s__Olsenella uli | Control | Dietary supplementation of FOS |
| s__Olsenella umbonata | Control | Dietary supplementation of FOS |
| s__Paenibacillus ihbetae | Control | Dietary supplementation of FOS |
| s__Paenibacillus stellifer | Control | Dietary supplementation of FOS |
| s__Petrimonas mucosa | Control | Dietary supplementation of FOS |
| s__Porphyromonas crevioricanis | Control | Dietary supplementation of FOS |
| s__Prevotella ruminicola | Control | Dietary supplementation of FOS |
| s__Proteiniphilum saccharofermentans | Control | Dietary supplementation of FOS |
| s__Slackia heliotrinireducens | Control | Dietary supplementation of FOS |
| s__Symbiobacterium thermophilum | Control | Dietary supplementation of FOS |
| s__Tannerella forsythia | Control | Dietary supplementation of FOS |
| s__Thermaerobacter marianensis | Control | Dietary supplementation of FOS |
| s__Thermobifida fusca | Control | Dietary supplementation of FOS |
| s__Thioalkalivibrio nitratireducens | Control | Dietary supplementation of FOS |
| s__Thioclava nitratireducens | Control | Dietary supplementation of FOS |
| s__Treponema azotonutricium | Control | Dietary supplementation of FOS |
| s__Treponema denticola | Control | Dietary supplementation of FOS |
| s__Treponema primitia | Control | Dietary supplementation of FOS |

**References**：

1. Zheng, X.J., Qiu, Y.P., Zhong, W., Baxter, S., Su, M.M., Li, Q., Xie, G.X., Ore, B.M., Qiao, S.L., Spencer, M.D., et al. (2013). A targeted metabolomic protocol for short-chain fatty acids and branched-chain amino acids. Metabolomics *9*, 818-827.

2. Li, G.L., Li, J., Kohl, K.D., Yin, B.F., Wei, W.H., Wan, X.R., Zhu, B.L., and Zhang, Z.B. (2019). Dietary shifts influenced by livestock grazing shape the gut microbiota composition and co-occurrence networks in a local rodent species. J Anim Ecol *88*, 302-314.
